# Impact of the COVID-19 Pandemic on Consultations and Diagnoses in Gastroenterology Practices in Germany

**DOI:** 10.3389/fmed.2021.684032

**Published:** 2021-05-31

**Authors:** Markus S. Jördens, Sven H. Loosen, Tobias Seraphin, Tom Luedde, Karel Kostev, Christoph Roderburg

**Affiliations:** ^1^Department of Gastroenterology, Hepatology and Infectious Diseases, University Hospital Düsseldorf, Medical Faculty of Heinrich Heine University Düsseldorf, Düsseldorf, Germany; ^2^Epidemiology, IQVIA, Frankfurt, Germany

**Keywords:** COVID-19, gastroenterologist, health care system Germany, health care system, consultations, diagnoses gastroenterology

## Abstract

The COVID-19 pandemic has been a major burden for healthcare systems worldwide and has caused multiple changes and problems in outpatient care. The aim of this study was to investigate the impact of the COVID-19 pandemic on consultations and diagnoses in gastroenterology practices in Germany. To this end, we retrospectively analyzed data from the Disease Analyzer database (IQVIA) using the International Classification of Diseases, 10th revision (ICD-10). We included all patients aged ≥18 years with at least one visit to one of 48 gastroenterology practices in Germany between April and September 2019 and April and September 2020. A total of 63,914 patients in the 2nd quarter of 2019, 63,701 in the 3rd quarter of 2019, 55,769 in the 2nd quarter of 2020, and 60,446 in the 3rd quarter of 2020 were included. Overall, a clear downward trend in the number of visits to gastroenterologists was observed in the 2nd quarter of 2020 compared to 2019 (−13%, *p* = 0.228). The decrease in consultations was particularly pronounced in patients >70 years of age (−17%, *p* = 0.096). This trend was evident for all gastrointestinal diagnoses except for tumors. Most notably, rates of gastrointestinal infections (−19%) or ulcers (−43%) were significantly lower in this period than in the same quarter of 2019. Reflecting the course of the pandemic, the differences between the 3rd quarter of 2020 and that of 2019 were less pronounced (−5%, *p* = 0.560). Our data show that the pandemic changed patients' behavior with respect to the health care system. Using the example of German gastroenterology practices, we show that the number of consultations as well as the number and range of diagnoses have changed compared to the same period in 2019.

## Introduction

Since its first description in December 2019, the coronavirus-2 (SARS-CoV-2) severe acute respiratory syndrome, later defined as the coronavirus disease 2019 (COVID-19), has spread around the globe and subsequently induced a severe pandemic often compared to the Spanish Flu at the beginning of the 20th century (1). As of February 10, 2021, the Johns-Hopkins University has recorded 106,965,011 cases and 2,342,808 cumulative deaths associated with the virus around the world (2). Nearly all nations have officially been affected by the pandemic, which naturally poses a major problem for healthcare systems worldwide.

The German outpatient care system is built on patient consultations with general practitioners, who then refer these patients to a specialist, e.g., a gastroenterologist, if medically indicated (3). A total of 1,438 gastroenterologists are currently working in the field of outpatient care in Germany (December 31, 2019) (4).

**Figure 1:**
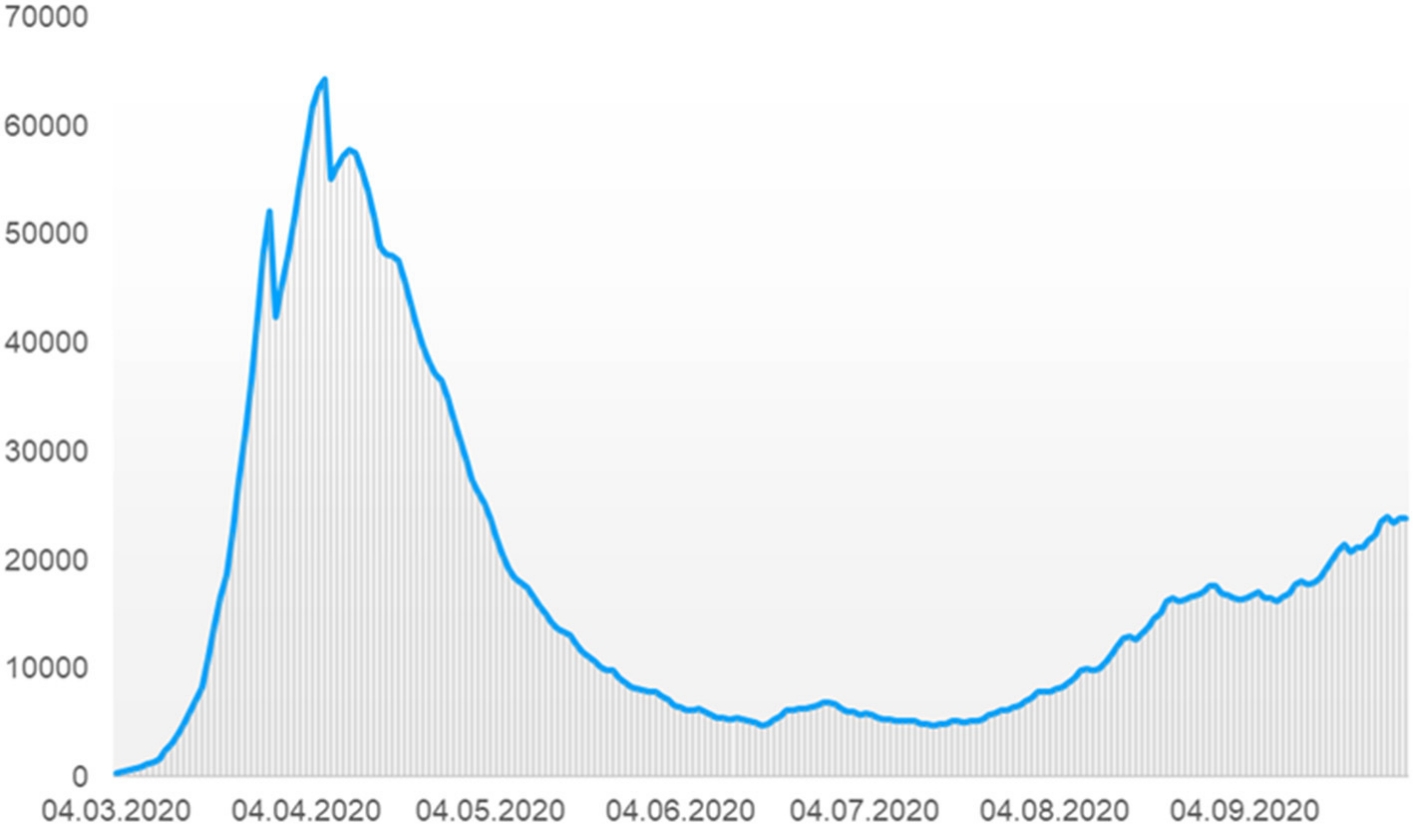
Number of people currently infected in Germany between March and September 2020 (adapted to the case numbers of the Robert Koch Institut [6]).

To prevent healthcare systems and, in particular, intensive care units from becoming overwhelmed by severe cases of COVID-19, governments around the globe implemented a range of non-pharmaceutical measures with different and changing levels of intensity (5). Accordingly, in the beginning of 2020, the German Federal Government imposed rigorous measures to contain the spread of the virus due to rising infection numbers (Figure 1) (6). This included, e.g., closures of bars and restaurants, schools and public playgrounds, as well as a recommendation for businesses to allow and facilitate remote working (7).

It seems likely that these measures as well as a general fear of becoming infected when using healthcare services have influenced the number and type of visits to physicians. In this retrospective analysis, using data from the Disease Analyzer database (IQVIA), we investigated the impact of the COVID-19 pandemic on consultations and diagnoses in gastroenterology practices in Germany. To the best of our knowledge, the present study is the first of its kind to address this important topic.

## Methods

This study used data from the Disease Analyzer database (IQVIA), full details of which have been published elsewhere (8). The Disease Analyzer database is composed of sociodemographic, diagnosis, and prescription data obtained in general and specialized practices in Germany. Diagnosis data are based on the German adaptation of the International Classification of Diseases, 10th revision (ICD-10), while prescription data are coded using the European Pharmaceutical Marketing Research Association (EphMRA) Anatomical Therapeutic Chemical (ATC) classification system. The quality of the data is assessed regularly by IQVIA based on a number of criteria (e.g., completeness of documentation and linkage between diagnoses and prescriptions). It has previously been found that the panel of practices included in the Disease Analyzer database is representative of general and specialized practices in Germany (8). Finally, this database has already been used for other studies investigating the impact of the Covid-19 pandemic on the healthcare system (9–11).

This retrospective study included all patients aged ≥18 years with at least one visit to one of 48 gastroenterology practices in Germany between April and September 2019 and April and September 2020. The database used has a coverage of 4.5% for gastroenterological practices, what means that 48 gastroenterological practices are included from 1,052 gastroenterological practices in Germany. From these 48 practices the complete information on consultations and diagnoses of patients followed in these practices is available.

The first outcome was the difference in the number of patients with at least one visit to these practices between the second and third quarters of 2019 and the second and third quarters of 2020. The number of patient visits per practice each quarter was compared for 2019 and 2020 using Wilcoxon tests. Comparisons were stratified by sex and age group. A *p*-value of <0.05 was considered statistically significant.

The second outcome was the number of patients with new diagnoses per practice, defined as diagnoses not previously documented in the database for a given patient. These diagnoses included the following categories: infectious and unspecified gastroenteritis and colitis (ICD-10: A09), esophagus diseases (ICD-10: K20-K22), ulcers (ICD-10: K25-K28), gastritis and duodenitis (ICD-10: K29), Crohn's disease and ulcerative colitis (ICD-10: K50, K51), liver diseases (ICD-10: K70-K77), diverticular disease (ICD-10: K57), anus and rectum diseases (ICD-10: k60-K62, K64), hernia (ICD-10: K40-K46), and cancer of digestive organs (ICD-10: C15-C26). In this study, one-sample Kolmogorov-Smirnov test was used to check whether the data (patient number per practice) are distributed normally or not. As there was an evidence that the data were not normally distributed. The number of patients with visits each quarter per practice was compared for 2019 and 2020 using non-parametric Wilcoxon signed-rank test. A *p*-value of <0.05 was considered statistically significant. The analyses were carried out using SAS 9.4 (SAS Institute, Cary USA).

## Results

### Study Population

This study included 63,914 patients in the 2nd quarter of 2019, 63,701 in the 3rd quarter of 2019, 55,769 in the 2nd quarter of 2020 and 60,446 in the 3rd quarter of 2020. The baseline characteristics are shown in the Table 1. There were significant differences in the age and sex distributions between 2019 and 2020, but these were very small in terms of absolute numbers.

**Table 1:** Age and sex characteristics of patients who visited 48 gastroenterology practices in the 2 and 3 quarters of 2019 and 2020.

**Patient group**	**Quarter 2/2019**	**Quarter 2/2020**	***P*-value**	**Quarter 3/2019**	**Quarter 3/2020**	***P*-value**
N	63,914	55,769		63,701	60,446	
Average age (mean, SD)	57.5 (16.3)	57.2 (16.2)	0.003	57.0 (16.4)	57.3 (16.5)	0.365
Age 18–40 years (%, 95 CI)	17.1 (16.8–17.4)	17.3 (17.0–17.6)	<0.001	18.0 (17.7–18.3)	17.6 (17.3–17.9)	0.369
Age 41–50 years (%, 95 CI)	12.5 (12.2–12.8)	12.2 (11.9–12.5)		12.5 (12.2–12.8)	12.1 (11.8–12.4)	
Age 51–60 years (%, 95 CI)	25.0 (24.7–25.3)	26.2 (25.8–26.6)		25.4 (25.1–25.7)	25.5 (25.2–25.9)	
Age 61–70 years (%, 95 CI)	22.4 (22.1–22.7)	22.2 (21.9–22.6)		21.6 (21.3–21.9)	22.1 (21.8–22.4)	
Age >70 years (%, 95 CI)	23.0 (22.7–23.3)	22.1 (21.8–22.4)		22.5 (22.2–22.8)	22.7 (22.4–23.0)	
Women (%, 95 CI)	53.5 (53.1–53.9)	52.5 (52.1–52.9)	<0.001	53.6 (53.2–54.0)	53.7 (53.3–54.1)	0.765
Men (%, 95 CI)	46.5 (46.1–46.9)	47.5 (47.1–47.9)		46.4 (46.0–46.8)	46.3 (45.9–46.7)	

### Changes in Diagnosis Frequencies and Diagnosis Distribution

Table 2 shows the differences by sex and age in the number of visits per practice between April and September 2020 and April and September 2019. The number of patients per practice was lower in the 2nd quarter of 2020 than in the 2nd quarter of 2019 (1,162 vs. 1,332, −13%, *p* = 0.228). These differences were observed for both women and men as well as different age groups. The most prominent differences (−17%) occurred in the age group >70 years. When comparing the 3rd quarter of 2020 with the 3rd quarter of 2019, the differences were smaller (1,259 vs. 1,327, −5%, *p* = 0.560), most likely reflecting the course of the pandemic and the mitigation of the restrictions imposed by the authorities.

**Table 2:** Differences by sex and age in the number of visits per practice in 48 German gastroenterology practices between April and September 2020 and April and September 2019.

**Patient group**	**Quarter 2/2019 (Mean, SD)**	**Quarter 2/2020(Mean, SD)**	**Difference in %**	***P*-value**	**Quarter 3/2019 (Mean, SD)**	**Quarter 3/2020(Mean, SD)**	**Difference in %**	***P*-value**
Total	1,332 (SD:646)	1,162 (SD:558)	−13	0.228	1,327 (SD:672)	1,259 (SD:636)	−5	0.560
Age 18–40 years	227 (SD:138)	201 (SD:128)	−11	0.278	239 (SD:150)	222 (SD:140)	−7	0.557
Age 41–50 years	166 (SD:92)	142 (SD:76)	−15	0.183	166 (SD:94)	152 (SD:89)	−8	0.391
Age 51–60 years	332 (SD:179)	304 (SD:160)	−9	0.393	337 (SD:189)	321 (SD:172)	−5	0.580
Age 61–70 years	298 (SD:144)	258 (SD:123)	−13	0.111	287 (SD:144)	278 (SD:140)	−3	0.727
Age >70 years	308 (SD:145)	257 (SD:116)	−17	0.096	299 (SD:144)	288 (SD:138)	−4	0.749
Women	713 (SD:358)	609 (SD:305)	−15	0.156	712 (SD:377)	676 (SD:354)	−5	0.620
Men	618 (SD:293)	552 (SD:258)	−11	0.268	616 (SD:299)	583 (SD:287)	−5	0.550

**Figure 2:**
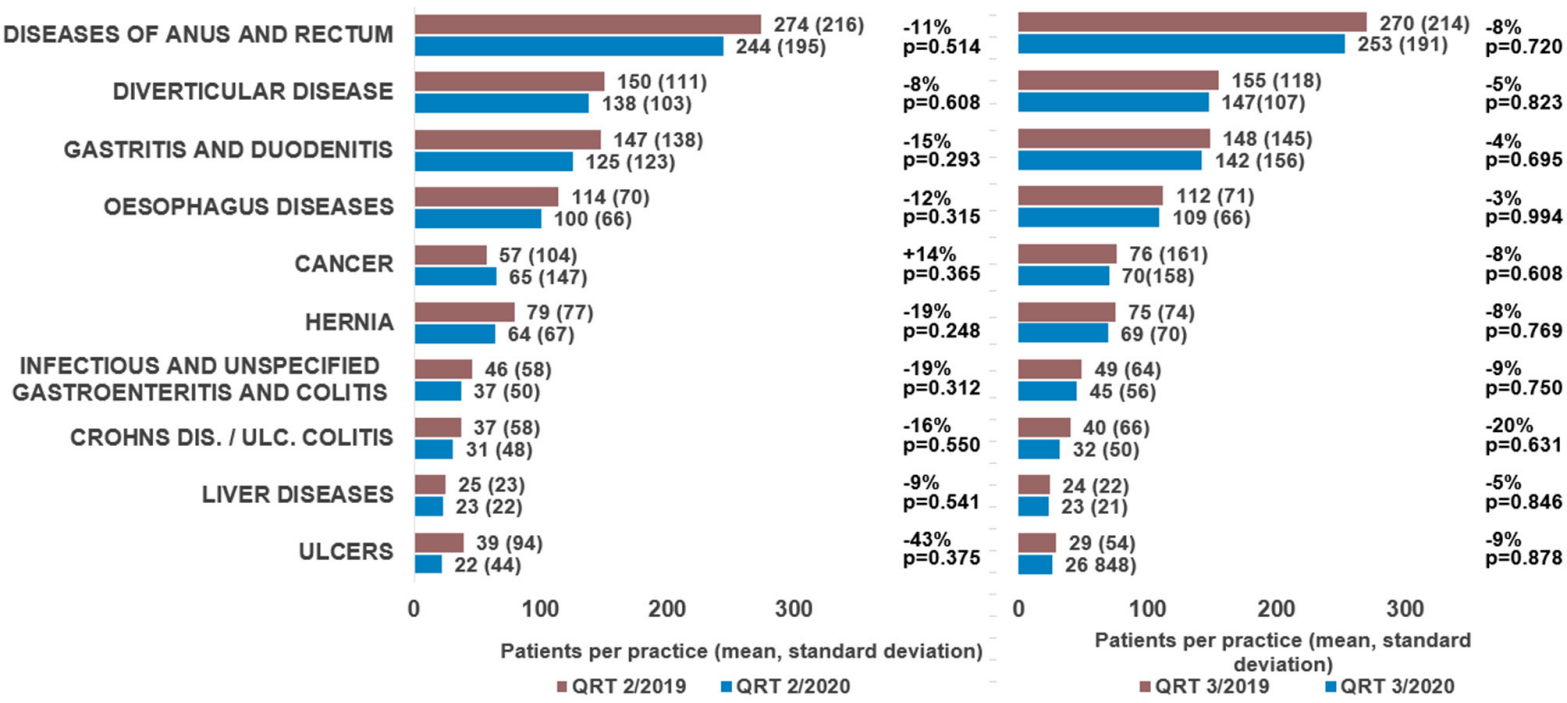
Differences in the number of new diagnoses per practice in 48 German gastroenterology practices between April and September 2020 and April and September 2019.

Along with the number of consultations, the number of defined diagnoses per practice decreased in the 2nd quarter of 2020 compared to the same period in 2019 (ulcers: −43%, hernia: −19%, infectious and unspecified gastroenteritis and colitis: −19%; Crohn's disease and ulcerative colitis: −16%, Figure 2). The only diseases demonstrating an increase were gastrointestinal cancers (+14%). Although none of the differences observed met the criteria for statistical significance, they still provide a clear picture of the changes caused by the first wave of the pandemic in Germany. Looking at the 3rd quarter, the differences were much smaller than in the 2nd quarter, providing further evidence that the pandemic played a role in causing the changes observed. Interestingly, only the number of patients with Crohn's disease and ulcerative colitis decreased further in the 3rd quarter of 2020 (−20%) with this effect actually slightly stronger than in the 2nd quarter.

## Discussion

The SARS Cov2 pandemic poses an unprecedented challenge to even well-established and well-funded healthcare systems, such as Germany's. During the first wave of the pandemic from March to May 2020, extensive efforts were made to prepare hospitals and the healthcare system as a whole for the arrival of larger numbers of patients infected with SARS-Cov2 (Figure 1). As part of this preparation, patients were advised to use the healthcare system in urgent cases only. In addition, many people were under the impression that using medical services might increase the risk of infection due to contact with doctors, nurses, or other patients. Overall, the number of outpatient treatments fell considerably, which resulted, for example, in an increased number of unrecognized cancer cases (12). In the present study, we analyzed the number of consultations in gastroenterology practices in Germany in the second and third quarters of 2019 and 2020. We observed a clear trend toward a reduction in office visits during (April to September) compared to the previous year. In particular, there was a 13% decrease in consultations in the 2nd quarter (April to June) of 2020 compared to the same period in 2019, and a much smaller decrease of 5% in the third quarter (July to September), which most likely reflects the course of the COVID-19 pandemic in Germany. This assumption is underlined by the fact that the decrease in consultations in the second quarter of 2020 was particularly noticeable in the group of people over 70 years of age (−17%), who are known to fall into the high-risk group for a severe or even fatal course of the COVID-19 disease due to their age and the underlying diseases that are common in this age group (13, 14). In this patient population it is particularly likely that uncertainty and fear of becoming infected in a physician's office led patients to cancel or postpone gastroenterological consultations. Interestingly, we observed a significant decrease in all gastroenterological disease patterns with the exception of cancer. It seems likely that patients with suspected malignancies were exempt from the general reduction in the number of patients accessing treatment and diagnostic services. Therefore, if a tumor was suspected, the patient was given further diagnostic clarification in the gastroenterological practice and the subsequent possibility of further inpatient treatment. We observed only a slight decrease in consultations in the 3rd quarter, which is most likely due to many patients catching up on visits that did not take place in the 2nd quarter.

One striking finding of our analysis is the significant reduction in gastrointestinal infections (−19%) and gastrointestinal ulcers (−43%) in Q2 2020 compared to Q2 2019. One explanation for the decrease in gastrointestinal infections could be that restaurants and bars were largely closed during the period and fewer people ate out, potentially leading to a lower risk of GI-infections (15, 16). In addition, contact and travel restrictions likely curtailed the spread of viral gastrointestinal infections (17, 18). Furthermore, the threshold for a physician visit due to gastrointestinal infection might have also been higher because of the COVID-19 pandemic, and more people would have opted to treat their symptoms at home without medical consultation. Similarly to gastrointestinal infections, the significant reduction in the number of patients diagnosed with ulcers (−43%) may be related to the fact that many patients did not present to a physician during the spring months when symptoms were only mild and spent longer trying to treat their symptoms themselves. Furthermore, the lockdown in spring 2020 and the associated partial closures of industry, stores and offices may also have led to a reduction in stress at work due to additional days off or increased home office hours, which could also have had a favorable effect on the development of gastrointestinal ulcerations as well as the potentially reduced transmission of *helicobacter pylori* due to the restriction of contacts (19–23).

Overall, our study provides strong evidence of a decrease in gastroenterology consultations during the COVID-19 pandemic compared to the previous year. Nevertheless, our study is subject to several limitations that are unavoidable within the study design for studies based on database analyses. First, misclassification or missing coding of individual diagnoses may have occurred due to the use of the ICS-10 coding system. Furthermore, the German Disease Analyzer database does not contain extensive laboratory or tissue analyses, nor information on lifestyle or socioeconomic status, and the data available do not allow disease severity to be analyzed. Finally, this study has a descriptive nature and no complex statistic methods like time-series analyses were used. In addition, we do not have any data on changes in diseases in hospitalized patients and therefore cannot exclude the possibility that some diseases that have decreased in the outpatient setting during the time periods we studied have increased in the inpatient setting during the same time period. Since the database includes only 4.5% of gastroenterology practices in Germany, conclusions can be susceptible to bias, and results might vary. Nevertheless, the database presents a relevant overview of the consultation behavior of the German population during the COVID-19 pandemic and shows an interesting shift in the number of gastroenterological physician visits in the pandemic year 2020 compared to the previous year.

## Conclusion

Our data suggest that the COVID 19 pandemic had an impact on the consultation behavior of gastroenterology patients in Germany.

## Data Availability Statement

The data that support the findings of this study are available on request from the corresponding author. Data included into this analysis represent highly sensitive medical data. It is directly against German (and European) law to publish such data in a way that would allow identifying individual patients (e.g. by providing different clinical values of one distinct patient). Data are available upon request from the Department of Gastroenterology, Hepatology and Infectious Diseases of the University Hospital Düsseldorf for researchers who meet the criteria for access to confidential data: Wissenschaft.Gastro@med.uni-duesseldorf.de.

## Ethics Statement

Ethical review and approval was not required for the study on human participants in accordance with the local legislation and institutional requirements. Written informed consent for participation was not required for this study in accordance with the national legislation and the institutional requirements.

## Author Contributions

MJ, SL, KK, and CR planed and conceptualized the work. Data collection and analysis was performed by KK. Data were verified by KK and SL. MJ, SL, TS, and CR wrote the original draft. TL and KK revised the draft. TL, KK, and CR supervised the work. CR had the project administration. All authors contributed to the article and approved the submitted version.

## Conflict of Interest

The authors declare that the research was conducted in the absence of any commercial or financial relationships that could be construed as a potential conflict of interest.
